# Evaluating the Machine Learning Models in Predicting Intensive Care Unit Discharge for Neurosurgical Patients Undergoing Craniotomy: A Big Data Analysis

**DOI:** 10.1007/s12028-025-02246-9

**Published:** 2025-05-06

**Authors:** Taghi Khaniyev, Efecan Cekic, Muhammet Abdullah Koc, Ilke Dogan, Sahin Hanalioglu

**Affiliations:** 1 Faculty of Engineering, Department of Industrial Engineering, Bilkent University, Ankara, Turkey; 2 National Magnetic Resonance Research Center, Bilkent University, Ankara, Turkey; 3 MIT Sloan School of Management, Massachusetts Institute of Technology, Cambridge, MA USA; 4 Faculty of Medicine, Department of Neurosurgery, Hacettepe University, Ankara, Turkey; 5 Faculty of Engineering, Department of Computer Engineering, Bilkent University, Ankara, Turkey; 6 Department of Neurosurgery, Brigham and Women’s Hospital, Harvard Medical School, Boston, MA USA

**Keywords:** Machine learning, Discharge, Intensive care unit, Neurosurgery, Craniotomy

## Abstract

**Background:**

Predicting intensive care unit (ICU) discharge for neurosurgical patients is crucial for optimizing bed sources, reducing costs, and improving outcomes. Our study aims to develop and validate machine learning (ML) models to predict ICU discharge within 24 h for patients undergoing craniotomy.

**Methods:**

The 2,742 patients undergoing craniotomy were identified from Medical Information Mart for Intensive Care dataset using diagnosis-related group and International Classification of Diseases codes. Demographic, clinical, laboratory, and radiological data were collected and preprocessed. Textual clinical examinations were converted into numerical scales. Data were split into training (70%), validation (15%), and test (15%) sets. Four ML models, logistic regression (LR), decision tree, random forest, and neural network (NN), were trained and evaluated. Model performance was assessed using area under the receiver operating characteristic curve (AUC), average precision (AP), accuracy, and F1 scores. Shapley Additive Explanations (SHAP) were used to analyze importance of features. Statistical analyses were performed using R (version 4.2.1) and ML analyses with Python (version 3.8), using scikit-learn, tensorflow, and shap packages.

**Results:**

Cohort included 2,742 patients (mean age 58.2 years; first and third quartiles 47–70 years), with 53.4% being male (*n* = 1,464). Total ICU stay was 15,645 bed days (mean length of stay 4.7 days), and total hospital stay was 32,008 bed days (mean length of stay 10.8 days). Random forest demonstrated highest performance (AUC 0.831, AP 0.561, accuracy 0.827, F1-score 0.339) on test set. NN achieved an AUC of 0.824, with an AP, accuracy, and F1-score of 0.558, 0.830, and 0.383, respectively. LR achieved an AUC of 0.821 and an accuracy of 0.829. The decision tree model showed lowest performance (AUC 0.813, accuracy 0.822). Key predictors of SHAP analysis included Glasgow Coma Scale, respiratory-related parameters (i.e., tidal volume, respiratory effort), intracranial pressure, arterial pH, and Richmond Agitation-Sedation Scale.

**Conclusions:**

Random forest and NN predict ICU discharge well, whereas LR is interpretable but less accurate. Numeric conversion of clinical data improved performance. This study offers framework for predictions using clinical, radiological, and demographic features, with SHAP enhancing transparency.

## Introduction

Neurosurgery patients undergoing craniotomy often constitute a significant portion of those requiring neurocritical care admission [1]. In many institutions, it is standard policy for the neurosurgery department to admit this broad spectrum of patients to the intensive care unit (ICU) postoperatively. This practice is due to the necessity for close monitoring and the high risk of postoperative complications, such as cerebral hemorrhage, edema, and seizures [2–4]. Therefore, postoperative monitoring and critical care of these patients continue to be essential to improving patient outcomes [5].

Optimizing ICU resource management is vital to maintain the availability of postoperative monitoring for incoming elective or urgent neurosurgery cases [6, 7]. Neurosurgical teams’ abilities to perform timely operations depends heavily on the availability of these ICU beds. ICU overcrowding delays these patients’ surgeries and other critical cases, which may cause transfers to external hospitals for critical care, morbidity, and even mortality [8].

The significant variability in individual recovery trajectories and complex clinical conditions in patients undergoing craniotomy make predicting ICU discharge times challenging [9]. Timely patient discharge from the ICU in these patients is crucial for optimizing ICU bed utilization, preventing patient overcrowding, and improving patient outcomes [10]. Thus, predicting patient discharge times can significantly alleviate the complications and reduce the costs [11–13]. This necessitates seamless coordination among neurosurgery, intensive care medicine, and other health care departments [14].

Current ICU discharge planning often relies on subjective or quasiobjective assessments by multidisciplinary clinical teams, which can be inconsistent, time consuming, and cause nontransparency among various departments [15]. This process involves evaluating various clinical indicators such as Acute Physiology and Chronic Health Evaluation scores, Glasgow Coma Scale (GCS) scores, pupil response, motor strength, respiratory patterns, hemodynamic stability, and comorbid conditions. Although these assessments are critical, they are often subjective and dependent on human factors such as individuals’ and ICU teams’ experiences. Therefore, they may not reliably predict discharge readiness with high accuracy [16, 17].

Machine learning (ML) algorithms present a promising solution to enhance medical decisions, discharge planning, and mortality prediction [9, 18–21]. Recent studies have shown the efficacy of ML models in predicting discharge times and identifying barriers to discharge in various sorts of patients [11, 22, 23]. These models can process extensive electronic medical record (EMR) data to provide timely and accurate predictions, supporting clinical decision-making and improving discharge processes [24–26]. In most studies predicting patient outcomes [11, 27], general/aggregate ML models (i.e., models trained with data from general patient populations) are preferred over specialized/cohort-specific models for two reasons: (1) the convenience of dealing with a single general model versus multiple cohort-specific models and (2) the possibility of learning patterns with explanatory power that are persistent across different cohorts. These aggregate models, however, usually come at the expense of reduced accuracy for some cohorts. Especially for cohorts that exhibit significantly different outcome behavior (e.g., cohorts that have significantly longer lengths of stay compared wtih the others), cohort-specific models are typically more accurate as long as there are a sufficient number of observations with which to train the ML model.

The ML models have shown significant promise in predicting critical outcomes across various medical domains, yet their application in predicting ICU discharge for neurosurgical patients undergoing craniotomy remains underexplored [28]. Accurate discharge predictions could enable better patient management, optimize ICU resource allocation, and reduce health care costs, addressing key challenges in neurocritical care.

This study aims to develop and validate ML models to predict ICU discharge within 24 h for neurosurgical patients undergoing craniotomy based on patient-day units. To achieve this, we used a large, multidimensional dataset, including diagnosis-related group (DRG) codes, International Classification of Diseases (ICD) codes, radiological reports, laboratory results, and daily clinical examinations. Textual data were systematically transformed into numerical values using manual grading and keyword-based approaches. By integrating diverse patient-specific features, we aim to identify critical factors influencing ICU discharge decisions and evaluate the predictive performance of different algorithms. Our findings aspire to provide actionable insights for improving neurocritical care practices and enhancing operational efficiency.

## Methods

### Data collection and preprocessing

The study was conducted and reported in adherence to the Transparent Reporting of a Multivariable Prediction Model for Individual Prognosis or Diagnosis guidelines. Our study used a publicly available EMR dataset, Medical Information Mart for Intensive Care (MIMIC-IV) [29, 30], containing postoperative ICU data from patients undergoing craniotomy. The MIMIC dataset was developed by the Laboratory for Computational Physiology at Massachusetts Institute of Technology in collaboration with Beth Israel Deaconess Medical Center and includes deidentified health care data of ICU patients [31].

**Fig. 1:**
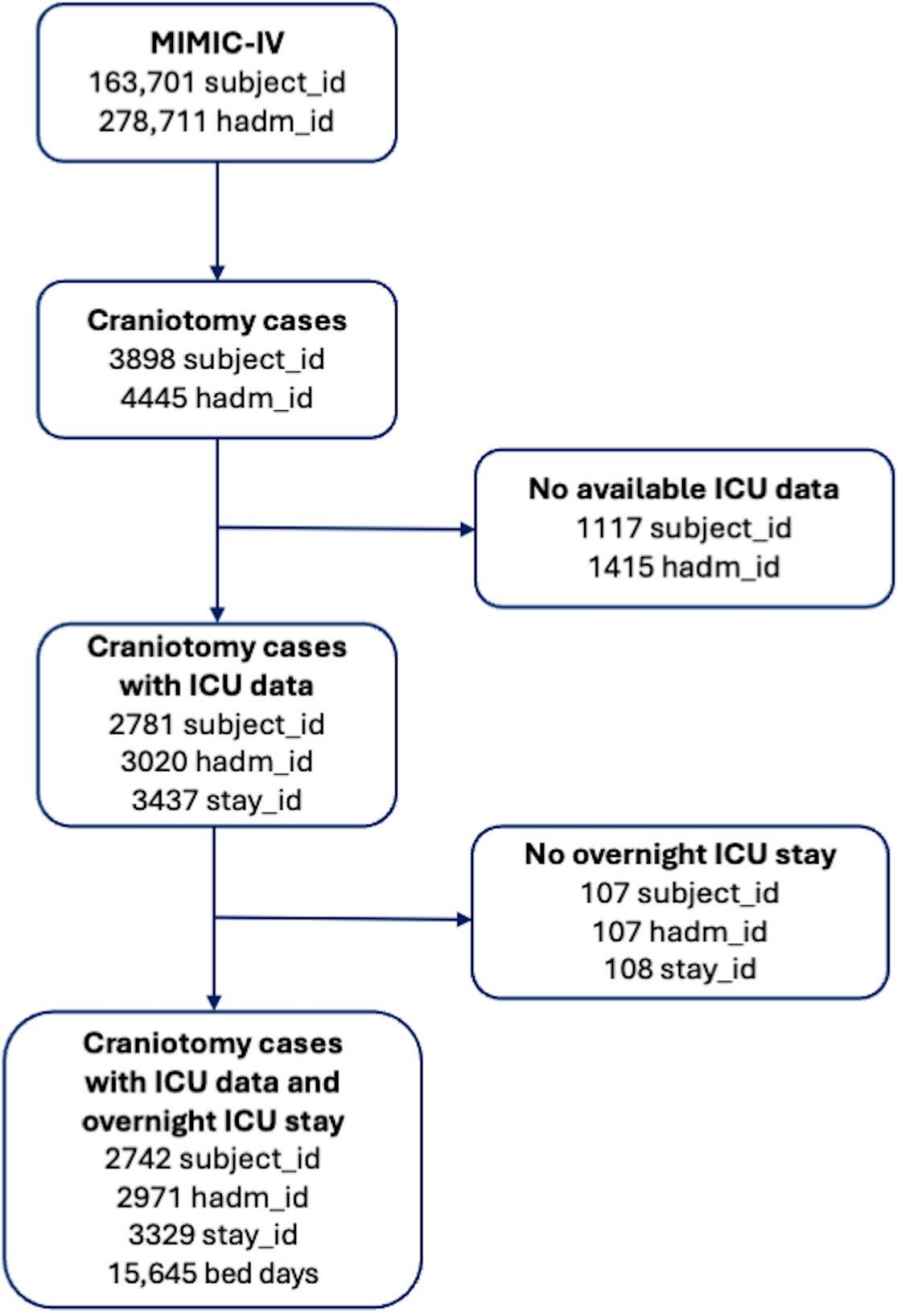
Patient selection process and inclusion–exclusion criteria flowchart. MIMIC-IV: Medical Information Mart for Intensive Care database, ICU: intensive care unit, subject_id: unique patient ID, hadm_id: unique hospital admission ID, stay_id: unique ICU stay ID

As detailed in Table 1, craniotomy cases were identified by a two-step approach using DRG and ICD procedural codes corresponding to cranial neurosurgical pathologies, such as brain tumors and aneurysms. We first screened the entire MIMIC-IV database by searching for the craniotomy and related keywords in the DRG codes. Thereafter, we extracted the list of ICD procedure codes (1,931 unique ICD codes) from all patients with at least one craniotomy-related DRG code. Finally, two neurosurgeons in our team screened these 1,931 ICD procedure codes and identified the codes that involved craniotomy (a total of 197 ICD codes). Patients with at least one of these 197 ICD codes during their hospitalization were included in our initial cohort. As detailed in Fig. 1, patients without ICU data or those who did not stay overnight in the ICU were excluded from the dataset. The final cohort was refined through a stepwise process, ensuring that the selected population consisted of patients undergoing craniotomy with at least one overnight ICU stay.

**Table 1:** The neurosurgery-relevant ICD codes among craniotomy patients in the MIMIC-IV cohort

Diagnosis	ICD Codes	Patient Count
Cerebral edema	3485, G936, S061X9A	1067
Compression of brain	3484, G935	696
Malignant neoplasm of brain	1983, 1911, C711, C7931, 1912, C712, 1913, C713, C710, 1918, 2375	669
Subdural hemorrhage	4321, 85,221, 85,220 S065X0A, I6203, S065X9A, S066X9A, I6200, 85,226	665
Cerebral hemorrhage	431, 430, I6201,99,811, 99,812, 4329, I611, I614, I615, I609, I618	595
Seizures	78,039, R569, 34,590, G4089, G40909, 3453, 34,550	437
Cerebral ınfarction	V1254, Z8673, 43,491, 43,411, I639, 99,702, 74,781, I67848, 43,310	433
Electrolyte Imbalance	2761, 2760, E870, 2,535E + 235	336
Benign neoplasm	2252, D320, 2250, 2396, 2251	323
Hemiplegia	34,290, 34,291, 34,292, G8191, G8194	278
Aphasia	7843, R4701	268
Hydrocephalus	3314, G911, G919	233
Dysphagia	R1310, 78,720	176
Cerebral aneurysm	4373, I671	159
Skull Fracture, Head Deformity or Traumatic Brain Injury	S0219XA, S020XXA, Z87820, V1552, 73,819, M952	147
Headache	7840, R51	113
Dysarthria	78,451, R471	101
Do not resuscitate status	V4986	98
Pituitary-related Conditions	D352, 2536	75
Encephalopathy	G9340, 34,839, 34,982	78
Intracranial abscess	3240, G060	52
Arteriovenous malformation of cerebral vessels	Q282	38

The dataset contained diverse clinical variables, including GCS scores, pupil response and size, motor strength and responsiveness, breathing patterns, comorbid disorders, and other relevant medical verbal information besides numerical data, such as laboratory results. To guarantee the quality and consistency of the data, we conducted typical preprocessing procedures, which involved addressing missing values, normalizing numerical values, and encoding categorical variables. Features with data sparsity (observed in fewer than 50 patients) were excluded to ensure reliable representation in the dataset and minimize overfitting.

The MIMIC-IV dataset includes semistructured data for clinical variables. We systematically extracted all possible variations of textual clinical descriptions and assigned numerical values. Because each variable had a finite set of possible alternatives, standardization was achieved efficiently without requiring complex neurolinguistic programming techniques. The examination results recorded as textual examination results were manually converted into numerical values through systematic grading by our clinical team. Each textual description was evaluated and assigned a clinical score on a scale reflecting best to worst outcomes, as detailed in Table 2, which comprehensively lists the clinical features used and their respective numerical scale values. The conversion process meant that the algorithm could uniformly read and analyze all clinical observations, hence improving the accuracy and reliability of the predictive ML model. Several of these scales were already in existence. Besides that, our expert neurosurgeons created others to transform verbal clinical information into numerical values, establishing a uniform measurement system for ML algorithms.

**Table 2:** Included non-numeric clinical indicators and corresponding numeric scales utilized in the study

Clinical Indicator	Description	Numeric Scale/Encoding
Orientation	Patient's awareness of person, place, time	0 = Disoriented, 1 = Oriented × 1, 2 = Oriented × 2, 3 = Oriented × 3
GCS—Eye Opening	Glasgow Coma Scale—Eye response	1 = No Response, 2 = To Pain, 3 = To Speech, 4 = Spontaneously
GCS—Verbal Response	Glasgow Coma Scale—Verbal response	1 = No Response, 2 = Incomprehensible sounds, 3 = Inappropriate Words, 4 = Confused, 5 = Oriented
GCS—Motor Response	Glasgow Coma Scale—Motor response	1 = No Response, 2 = Abnormal Flexion, 3 = Abnormal Extension, 4 = Withdraws, 5 = Localizes Pain, 6 = Obeys Commands
Pupil Response	Reaction to light	0 = Non-reactive, 1 = Sluggish, 2 = Brisk
Pupil Size	Pupil diameter	0 = < 6 mm, 1 = ≥ 6 mm
Strength (Right Arm, Right Leg, Left Arm, Left Leg)	Muscle strength assessment	0 = No movement, 1 = Movement but not against gravity, 2 = Lifts against gravity, no resistance, 3 = Some resistance, 4 = Normal strength
Richmond-RAS Scale	Sedation and agitation levels	-5 = Unarousable, -4 = Deep sedation, -3 = Moderate sedation, -2 = Light sedation, -1 = Awakens to voice, 0 = Alert and calm, + 1 = Anxious, + 2 = Frequent nonpurposeful movement, + 3 = Pulls or removes tubes, + 4 = Combative
Speech	Clarity and coherence of speech	0 = Mute, 1 = Garbled, 2 = Dysarthric, 3 = Normal
Cough Reflex	Ability to cough	0 = Absent, 1 = Impaired, 2 = Intact
Gag Reflex	Ability to gag	0 = Absent, 1 = Impaired, 2 = Intact
Level of Consciousness	Alertness and responsiveness	0 = Unresponsive, 1 = Awake but unresponsive, 2 = Lethargic, 3 = Arouse to Pain, 4 = Arouse to Voice, 5 = Alert, 6 = Oriented
Motor	Motor response	0 = No response, 1 = Abnormal extension, 2 = Abnormal flexion, 3 = Localizes pain, 4 = Withdraws, 5 = Obeys commands, 6 = Normal
Seizure Activity	Occurrence of seizures	0 = No, 1 = Yes
Pain Management	Type of pain management	0 = None, 1 = PO Medication, 2 = SC Injection, 3 = IV Push, 4 = PCA
Pain Present	Presence of pain	0 = No, 1 = Yes
Lung Sounds	Quality of lung sounds	0 = Diminished, 1 = Clear
Cough Effort	Strength of cough	0 = Weak, 1 = Strong
Respiratory Pattern	Breathing pattern	0 = Abnormal, 1 = Normal
Respiratory Effort	Effort of breathing	0 = Labored, 1 = Normal
O_2_ Delivery Device(s)	Type of oxygen delivery device	0 = None, 1 = Nasal cannula, 2 = Face mask, 3 = High flow nasal cannula, 4 = Non-rebreather mask, 5 = CPAP mask
Heart Rhythm	Heart rhythm type	0 = Asystole, 1 = Normal Sinus Rhythm, 2 = Sinus Tachycardia, 3 = Sinus Bradycardia, 4 = Atrial Fibrillation, 5 = Other
Activity / Mobility (JH-HLM)	Level of activity and mobility	0 = Bedrest, 1 = Sit at edge of bed, 2 = Transfer to chair, 3 = Walk 10 + Steps, 4 = Walk 25 + Feet, 5 = Walk 250 + Feet
Anti Embolic Device Status	Use of anti-embolic devices	0 = Off, 1 = On
Therapeutic Bed	Type of therapeutic bed used	0 = None, 1 = Any
Medication Usage*	Use of medications	0 = No, 1 = Yes
Intubation	Indicator of whether the patient is intubated	0 = No, 1 = Yes
On ventilation support	Indicator of whether the patient is on ventilation support	0 = No, 1 = Yes
Surgical Same Day Admission	Admission for same-day surgery	0 = No, 1 = Yes
Observation Admit	Admission for observation	0 = No, 1 = Yes
Emergency/Urgent	Emergency/Urgent admission status	0 = No, 1 = Yes
Elective	Elective admission status	0 = No, 1 = Yes

^*^Medications: Propofol, Heparin Sodium, Fentanyl, Nicardipine, Norepinephrine, Levetiracetam, Labetalol, Metoprolol, Midazolam, Lorazepam, Nicardipine, Nitroprusside, Mannitol, Diltiazem, Epinephrine, Dopamine

Table 3 details the laboratory results and other numerical values integrated into our ML models. The input features encompass not only these numerically encoded clinical indicators, but also additional variables related to patient demographics (e.g., age, sex) and operational parameters (e.g., time since admission, type of admission). These features provide a comprehensive dataset, enabling the model to capture many factors influencing patient outcomes.

**Table 3:** Numerical features, feature descriptions, and reference ranges used in ML models

Feature Name	Description	Referance range
Sodium (serum)	Serum sodium concentration	135–145 mmol/L
Potassium (serum)	Serum potassium concentration	3.5–5.0 mmol/L
Chloride (serum)	Serum chloride concentration	96–106 mmol/L
Glucose (serum)	Serum glucose concentration	70–100 mg/dL (fasting)
HCO3 (serum)	Serum bicarbonate concentration	22–28 mmol/L
Creatinine (serum)	Serum creatinine concentration	0.6–1.2 mg/dL (varies by muscle mass and age)
Anion gap	Difference between measured cations and anions	8–16 mEq/L
BUN	Blood urea nitrogen level	7–20 mg/dL
Magnesium	Serum magnesium concentration	1.7–2.2 mg/dL
Phosphorous	Serum phosphorus concentration	2.5–4.5 mg/dL
Hemoglobin	Concentration of hemoglobin in blood	13.8–17.2 g/dL (male), 12.1–15.1 g/dL (female)
Hematocrit (serum)	Proportion of red blood cells in blood	40.7–50.3% (male), 36.1–44.3% (female)
Platelet Count	Number of platelets in blood	150,000–450,000/μL
WBC	White blood cell count	4,500–11,000/μL
WBC > 12	Elevated white blood cell count (greater than 12 × 10^9/L)	> 12 × 10^9/L
WBC < 4	Low white blood cell count (less than 4 × 10^9/L)	< 4 × 10^9/L
PTT	Partial thromboplastin time	25–35 s
Prothrombin time	Time for blood to clot	11–13.5 s
INR	International normalized ratio	0.8–1.1
PH (Arterial)	Arterial blood pH	7.35–7.45
Arterial CO2 Pressure	Partial pressure of carbon dioxide in arterial blood	35–45 mmHg
Lactic Acid	Serum lactic acid concentration	0.5–2.2 mmol/L
AST	Aspartate transaminase enzyme level	10–40 IU/L
ALT	Alanine transaminase enzyme level	7–56 IU/L
Albumin	Serum albumin concentration	3.5–5.0 g/dL
Arterial O2 Saturation	Oxygen saturation in arterial blood	95–100%
Total Bilirubin	Total bilirubin concentration in blood	0.1–1.2 mg/dL
Troponin-T	Cardiac troponin-T level	< 0.01 ng/mL
CK-MB	Creatine kinase-MB level	< 5 ng/mL
Intra Cranial Pressure	Pressure inside the skull	9–20 cmH_2_O
Cerebral Perfusion Pressure	Mean Arterial Pressure- Intracranial Pressure	60–80 mmHg
PEEP set	Positive end-expiratory pressure setting	5–10 cm H_2_O
Minute Volume	Volume of gas inhaled/exhaled per minute	5–8 L/min
Mean Airway Pressure	Average pressure in the airways during a respiratory cycle	10–20 cm H_2_O
Apnea Interval	Interval of no breathing	should not occur
Tidal Volume	Volume of air moved per breath	500–600 mL
Heart Rate	Number of heartbeats per minute	60–100 bpm
Non Invasive Blood Pressure mean	Mean blood pressure measured non-invasively	70–100 mmHg
Arterial Blood Pressure mean	Mean arterial blood pressure	70–100 mmHg
Temperature Fahrenheit	Body temperature in Fahrenheit	98.6°F (37 °C)
Temperature Low	Low body temperature	< 97°F (< 36.1 °C)
Temperature High	High body temperature	> 100.4°F (> 38.3 °C)
Culture result positive	Positive microbiological culture result	N/A
Respiratory Rate > 20	Elevated respiratory rate (greater than 20 breaths per minute)	> 20 breaths/min
Arterial CO2 Pressure < 32	Low arterial CO2 pressure (less than 32 mmHg)	< 32 mmHg
Arterial O2 Pressure < 80	Low arterial oxygen pressure (less than 80 mmHg)	< 80 mmHg
O2 saturation pulseoxymetry < 92	Low oxygen saturation by pulse oximetry (less than 92%)	< 92%
Time since admission	Time elapsed since hospital admission	N/A (days)
Time since last ICU hospitalization	Time elapsed since last ICU admission	N/A (days)

A keyword-based screening approach was applied to radiology reports, followed by multiple refinement iterations to enhance classification accuracy. To correctly interpret negative expressions (e.g., “no midline shift”), a negation detection step was implemented to avoid misclassification. This involved recognizing phrases that explicitly indicated the absence of a condition (e.g., “no displacement of midline” or “midline within normal limits”). Table 4 outlines the positive and negative keywords used in this classification process. Positive keywords (e.g., “midline shift present,” “shift of midline structures”) were matched against report texts to identify abnormalities. In contrast, negative keywords (e.g., “no midline shift,” “midline intact”) were used to exclude false-positive results. This methodology enabled identifying radiological features with high classification accuracy (verified by manual reviews), ensuring reliable integration into the predictive model.

**Table 4:** Positive and negative keywords for radiological reports evaluation

Category	Positive Keywords	Negative Keywords	Count (%) of Positive Observaitons
Midline Shift	midline shift present, midline deviation, shift of X mm, asymmetry in midline, displaced midline, shift of midline structures, shift of normally midline structures	no midline shift, midline intact, midline normal, no displacement of midline, midline stable, midline within normal limits, no shift of normally midline structures, without shift of normally midline structures, without midline shift identified, without evidence of midline shift	2993 (19.1%)
Brain Edema	brain edema, cerebral swelling, diffuse swelling, significant edema, edematous parenchyma	no edema, no swelling, brain parenchyma normal, no cerebral edema, no cerebral swelling	63 (0.4%)
Subdural Hematoma	subdural hematoma, SDH, subdural collection, subdural fluid	no subdural hematoma, no SDH, no subdural collection, no subdural fluid, subdural space clear	2482 (15.8%)
Epidural Hematoma	epidural hematoma, EDH, epidural collection	no epidural hematoma, no EDH, no epidural collection	218 (1.3%)
Aneurysm	aneurysm, vascular dilation, arterial aneurysm	no aneurysm, normal vasculature, no vascular abnormality	869 (5.55%)
Tumor	brain tumor, malignant lesion, mass effect, neoplasm	no tumor, no mass, normal brain tissue, no neoplasm detected	2218 (14.1%)
Mass Effect	mass effect, compression, displacement, brainstem compression, ventricular compression	no mass effect, no compression, normal ventricle position	2842 (18.1%)
Herniation	herniation, uncal herniation, tonsillar herniation, brain herniation	no herniation, no evidence of herniation, normal intracranial alignment	1232 (7.8%)
Hydrocephalus	ventricular enlargement, dilated ventricles, hydrocephalus	no hydrocephalus, ventricles normal, ventricular size within normal limits	763 (4.8%)
Infarction or Ischemia	ischemia, cerebral infarction, restricted diffusion, acute stroke	no infarction, no ischemia, no restricted diffusion	291 (1.8%)
Hemorrhage	intracranial hemorrhage, ICH, bleeding, parenchymal bleed	no intracranial hemorrhage, no ICH, no bleeding	3230 (20.6%)
Infection or Abscess	abscess, infection, cerebral abscess, infected lesion	no abscess, no infection, no evidence of cerebral abscess	421 (2.6%)

To ensure consistency in patient data, all clinical features used in the analysis were derived from the most relevant recorded values before discharge. Specifically, the first type of features include binary features that indicate whether an event occurred within the last 24 h (e.g., high temperature, abnormal laboratory results). The feature value is set to 1 (true) if any abnormal value was recorded within the last 24 h, regardless of the most recent measurement. If no abnormal values were recorded in this period, the feature value is set to 0 (false). The second type of features include binary features that indicate whether an event ever occurred since the admission (e.g., culture positive test result, entry of an ICD code). The feature value is set to 1 (true) if the event occurred at some point between admission and the time of prediction and 0 (false) otherwise. Finally, the third type includes continuous features indicating the latest recorded measurement (e.g., laboratory values, vital signs). Only the latest value is used if multiple values were available between admission and the prediction time. Table 5 details the timing criteria for each clinical feature, including laboratory values, GCS scores, medication administration, and oxygen delivery devices.

**Table 5:** Timing of the record used for feature generation

Fixed Columns	Last 24 Hours	Ever Recorded During Admission	Last Recorded			
			Lab	Clinical	Device and Drugs	Radiology Reports
Gender – Female	Temperature Low	Culture Result Positive	Sodium	Orientation	O2 Delivery Device(s)	Midline Shift
Age 18–35	Temperature High	Infectious Disease	Potassium	Gcs—Eye Opening	Inspired O2 Fraction	Brain Edema
Age 35–50	Heart Rate > 90	***ICD Procedure Codes***	Chloride	Gcs—Verbal Response	Minute Volume	Subdural Hematoma
Age 50–65	Respiratory Rate > 20	0159—Other excision or destruction of lesion or tissue of brain	Glucose	Gcs—Motor Response	Mean Airway Pressure	Epidural Hematoma
Age > 65	Arterial Co2 Pressure < 32	0131—Incision of cerebral meninges	Hco3	Pupil Size Right	Apnea Interval	Aneurysm
Emergency Admission	White Blood Count > 12	0212—Other repair of cerebral meninges	Creatinine	Pupil Size Left	Tidal Volume (observed)	Tumor
Surgical Same Day Admission	White Blood Count < 4	8841—Arteriography of cerebral arteries	Anion Gap	Pupil Response Right	Heart Rhythm	Mass Effect
Observation Admit	Arterial O2 Pressure < 80	00C40ZZ—Extirpation of Matter from Intracranial Subdural Space, Open Approach	Bun	Pupil Response Left	Par-respiration	Herniation
Urgent	O2 Saturation Pulseoxymetry < 92	00B70ZZ—Excision of Cerebral Hemisphere, Open Approach	Magnesium	Strength R Arm	Par-consciousness	Hydrocephalus
Elective	Is Intubated	0151—Excision of lesion or tissue of cerebral meninges	Phosphorous	Strength R Leg	Par-oxygen Saturation	Infarction Or Ischemia
	On Ventilation Support	0139—Other incision of brain	Hemoglobin	Strength L Arm	Anti Embolic Device Status	Hemorrhage
		0124—Other craniotomy	Hematocrit	Strength L Leg	Activity / Mobility	Cerebral Atrophy
		00B00ZZ—Excision of Brain, Open Approach	Platelet Count	Speech	Propofol	Infection or Abscess
		0206—Other cranial osteoplasty	Ptt	Cough Reflex	Heparin Sodium (prophylaxis)	
		00B10ZZ—Excision of Cerebral Meninges, Open Approach	Prothrombin Time	Gag Reflex	Fentanyl	
		00N00ZZ—Release Brain, Open Approach	INR	Level Of Consciousness	Nicardipine	
		0331—Spinal tap	Ph (arterial)	Motor L Arm	Norepinephrine	
		009630Z—Drainage of Cerebral Ventricle with Drainage Device, Percutaneous Approach	Lactic Acid	Motor L Leg	Levetiracetam (keppra)	
		00B70ZX—Excision of Cerebral Hemisphere, Open Approach, Diagnostic	AST	Motor R Arm	Labetalol	
		***ICD Diagnostic Categories***	ALT	Motor R Leg	Metoprolol	
		Cerebral Edema	Albumin	Ru Strength/movement	Midazolam (versed)	
		Compression of Brain	Arterial O2 Saturation	Lu Strength/movement	Lorazepam (ativan)	
		Malignant Neoplasm of Brain	Total Bilirubin	Rl Strength/movement	Nicardipine 40 mg/200	
		Subdural Hemorrhage	Troponin-T	Ll Strength/movement	Nitroprusside	
		Seizures	CK-MB	Seizure Activity	Mannitol	
		Hemiplegia	Intra Cranial Pressure	Pain Management	Diltiazem	
		Aphasia	Cerebral Perfusion Pressure	Pain Present	Epinephrine	
		Dysphagia		Richmond-ras Scale	Dopamine	
		Cerebral Aneurysm		Rll Lung Sounds		
		Other Conditions Of Brain		Lll Lung Sounds		
		Headache		Rul Lung Sounds		
		Dysarthria		Lul Lung Sounds		
		Pituitary-related Conditions		Cough Effort		
		Encephalopathy		Cough Type		
		Intracranial Abscess		Respiratory Pattern		
		Arteriovenous Malformation Of Cerebral Vessels		Respiratory Effort		
				Breathing Pattern/effort		
				Current Dyspnea Assessment		

As demonstrated in Table 6, a comparative analysis was conducted to distinguish characteristics between the patients with and without overnight stays. This table highlights key differences in demographics, diagnoses, and procedural data, providing insights into factors influencing prolonged ICU admissions.

**Table 6:** Comparison of demographics, the most frequent neurosurgery-relevant ICD procedures and diagnoses between patients with and without at least one overnight ICU stay (for Sex and Age subject_id level comparison is made (107 vs. 2742), for the procedure_ids and diagnosis_ids, hadm_id level comparison is made (107 vs. 2971))

Feature	Value	Without Overnight ICU Stay [Count (%)] (n = 107)	With Overnight ICU Stay [Count (%)] (n = 2742)	*P*-value
Sex	Female	43 (40.1%)	1278 (46.6%)	0.191
Age	Mean ± SD	59.8 ± 17.0	58.2 ± 17.1	0.332
# 1 Procedure ICD	0159—Other excision or destruction of lesion or tissue of brain	25 (23.1%)	688 (20.7%)	0.960
# 2 Procedure ICD	0131—Incision of cerebral meninges	15 (13.9%)	460 (13.8%)	0.680
# 3 Procedure ICD	0212—Other repair of cerebral meninges	14 (13.0%)	310 (9.3%)	0.380
# 4 Procedure ICD	0151—Excision of lesion or tissue of cerebral meninges	8 (7.4%)	162 (4.9%)	0.367
# 5 Procedure ICD	00B70ZZ—Excision of Cerebral Hemisphere, Open Approach	6 (5.6%)	167 (5.0%)	0.995
# 6 Procedure ICD	0124—Other craniotomy	5 (4.6%)	112 (3.4%)	0.631
# 7 Procedure ICD	0139—Other incision of brain	5 (4.6%)	118 (3.5%)	0.716
# 8 Procedure ICD	00U20KZ—Supplement Dura Mater with Nonautologous Tissue Substitute	4 (3.7%)	139 (4.2%)	0.649
# 9 Procedure ICD	00C70ZZ—Other incision of brain	2 (1.9%)	27 (0.9%)	0.312
# 10 Procedure ICD	00N00ZZ—Release Brain, Open Approach	0 (0)	70 (2.1%)	0.175
# 1 Diagnosis	Cerebral edema	30 (27.8%)	1057 (31.8%)	0.108
# 2 Diagnosis	Convulsions	22 (20.4%)	467 (14.0%)	0.178
# 3 Diagnosis	Malignant neoplasm of brain	22 (20.4%)	656 (19.7%)	0.709
# 4 Diagnosis	Compression of brain	19 (17.6%)	690 (20.7%)	0.186
# 5 Diagnosis	Cerebral hemorrhage	18 (16.7%)	507 (15.2%)	0.947
# 6 Diagnosis	Subdural hemorrhage	13 (12.0%)	515 (15.5%)	0.162
# 7 Diagnosis	Cerebral infarction	12 (11.1%)	463 (13.9%)	0.219
# 8 Diagnosis	Skull Fracture, Head Deformity or Traumatic Brain Injury	12 (11.1%)	142 (4.3%)	**0.0027**
# 9 Diagnosis	Hemiplegia	11 (10.2%)	276 (8.3%)	0.729
# 10 Diagnosis	Aphasia	10 (9.3%)	265 (8.0%)	0.879

### ML models

Because our prediction task (whether a patient will be discharged within 24 h) is a binary classification problem, we tested four different ML algorithms: logistic regression (LR), decision trees (DT), random forest (RF), and feedforward neural networks (NN). For the details of the algorithm, the reader is referred to Khaniyev et al. (2025) [32]. Of the four algorithms tested, LR and DT are considered interpretable models because they make it possible, due to their simple structure, to interpret why a specific prediction is being made based on the values of the input features. RF and NN, on the other hand, are more complex “black-box” methods that do not lend themselves to simple interpretations of the predictions. There has been, however, a growing body of literature proposing tools to “explain” the predictions of such black-box models in terms of the values of input features (e.g., Local Interpretable Model-agnostic Explanations [33], Shapley Additive Explanations [SHAP] [34], etc.). The interpretability advantage of LR and DT usually comes at the expense of model accuracy compared to black-box models such as RF and NN. In our Results section, we will briefly analyze how the tradeoff between interpretability versus accuracy plays out. The dataset was split into training (70%), validation (15%), and test (15%) samples to train, tune hyperparameters, and evaluate the models’ performance. The performance metrics used for comparison were the area under the receiver operating characteristic curve (AUC), accuracy, average precision (AP), and F1 scores. Different values for hyperparameters for each method (i.e., regularization type and strength for LR; number of layers/nodes and regularization strength for NN; maximum depth of a tree, and minimum number of samples required for each leaf for DT and RF, and number of trees/estimators for RF) were tried and the hyperparameter combination that provides the best results in the validation sample were selected for each method. During hyperparameter optimization, multiple performance metrics were evaluated, including AUC, accuracy, AP, and F1 scores.

The F1 score is a performance metric used in classification tasks, particularly in imbalanced datasets. It is the harmonic mean of precision (positive predictive value) and recall (sensitivity), ensuring a balance between false-positive and false-negative results. The F1 score ranges from 0 to 1, in which a higher value indicates better model performance by balancing precision and recall. Generally, an F1 score above 0.7 is considered strong in many practical applications, although the threshold for “strong” performance depends on the task’s complexity and class balance. A score below 0.5 typically indicates limited predictive power, especially in balanced datasets, but it may still hold value in highly imbalanced or complex prediction tasks. The F1 score is calculated using the following formula: F1 score = 2 × ([precision × recall] / [precision + recall])​. The final model selection was based on AUC, which comprehensively measures model performance across different decision thresholds.

Our model employs a sliding window approach, in which the observation window consists of all prior data up to the prediction time, and the prediction window (the subsequent 24 h from the prediction time) is for which we predict if the patient will be discharged from ICU. Each prediction is based on a patient-day unit, in which each day of ICU stay is treated as an independent observation. The sliding window approach enables continuous and dynamic risk assessment by shifting the prediction window in daily increments over time. This method allows the model to generate new predictions at regular intervals (e.g., daily), ensuring that patient data are continuously updated and assessed [35].

### Feature importance (SHAP analysis)

For simple interpretable models like LR or DT, it is relatively easy to deduce which features are contributing to the prediction made for an observation. It is not, however, as straightforward to understand how much each feature contributed to a given prediction of black-box models such as NN and RF, due to their complex nature. A large body of literature on explainable artificial intelligence [30, 33] has recently focused on alleviating the problem of explaining the predictions of black-box models. The most prominent among the explainable artificial intelligent methods is SHAP, which provides, for a given observation, how much of the predicted value is attributable to each feature. We conducted the SHAP analysis to explain the predictions of the RF model because it had the highest predictive power among the algorithms tested. SHAP values obtained for each feature of each observation are, in turn, combined to derive an overall feature importance indicating how each clinical feature influenced the RF model’s predictions for ICU discharge. Each dot represents a single observation in the SHAP summary plot. Features are color-coded to represent their values: red denotes higher feature values, whereas blue denotes lower values. The horizontal axis indicates the direction and magnitude of each feature’s impact on discharge likelihood. Features on the right with positive SHAP values increase the possibility of discharge, whereas features on the left with negative SHAP values decrease the possibility of discharge. Suppose a feature is predominantly red on the right side (e.g., “GCS – Verbal Response” [0: NO, 1: YES]); it suggests that higher values (1: YES) of this feature are favorable (i.e., higher values increase the likelihood of discharge). Conversely, if a feature is predominantly blue on the right side (e.g., O2 Delivery Device[s] [0: NO, 1: YES]), it suggests that lower values (0: NO) of this feature are favorable (i.e., lower values increase the likelihood of discharge).

### Statistical analysis

We performed all statistical analyses with R (version 4.2.1) and ML training/analyses with Python (version 3.8) using the scikit-learn, tensorflow, and shap libraries. Descriptive statistics for parametric continuous variables were presented as means and standard deviations, whereas nonparametric continuous variables were summarized using medians, first and third quartiles. Categorical variables were summarized as numbers (percentages, [*n* {%}]). Group comparisons were conducted using independent t-tests for normally distributed variables and Mann–Whitney *U*-tests for nonnormally distributed variables. When appropriate, categorical variables were compared using *χ*^2^ or Fisher’s exact tests. A *p* value of less than 0.05 was considered statistically significant.

## Results

### Patient cohort and data characteristics

**Fig. 2:**
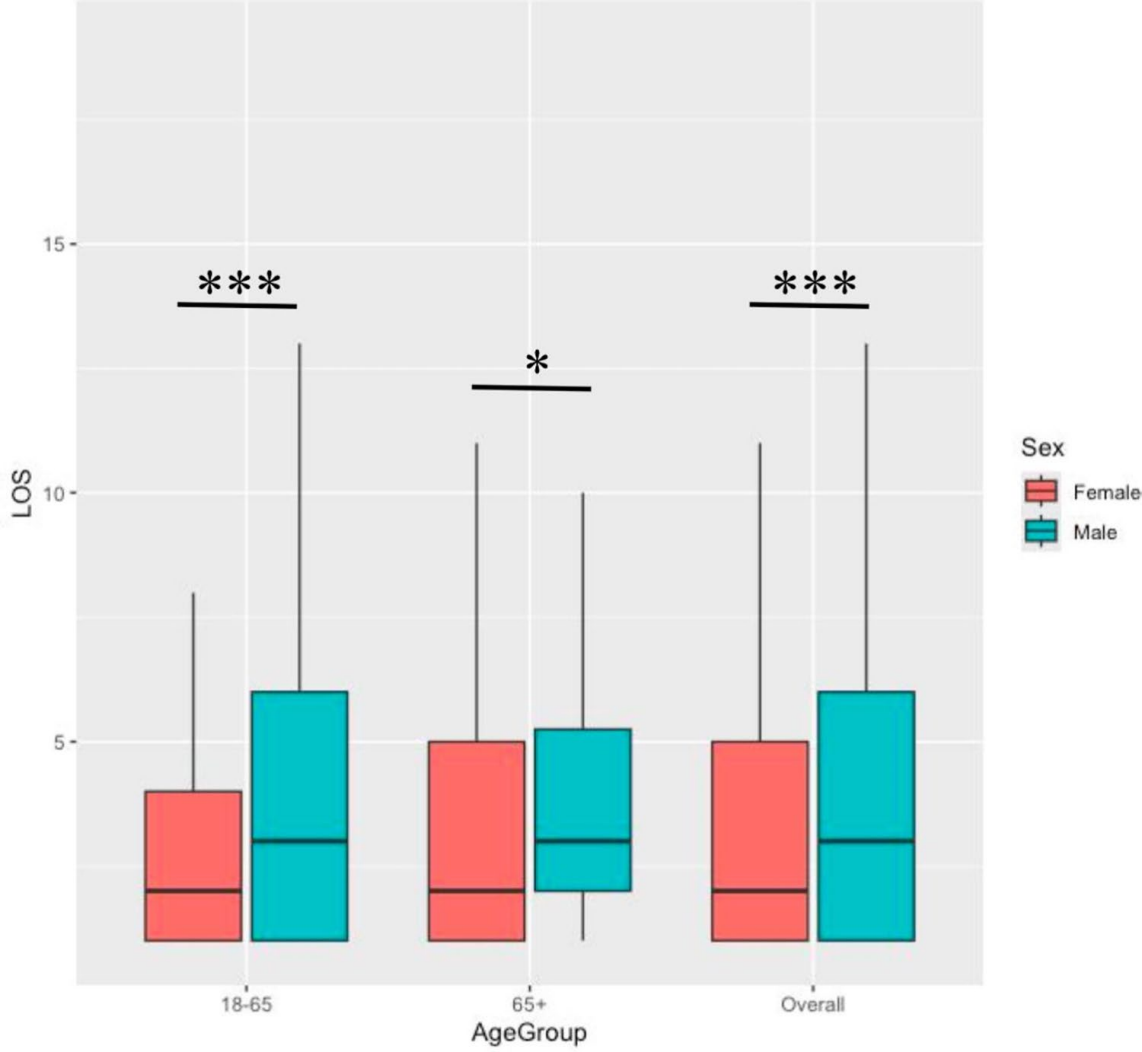
Boxplot of Length of Stay (LOS) in ICU by sex and age group

After applying inclusion criteria and data preprocessing (Fig. 1), 163,701 patients (278,711 hospital admissions) were initially screened from the MIMIC-IV database. Of these, 3,898 craniotomy cases (4,445 admissions) were identified. Following exclusions, that is, 1,117 patients without ICU data and 107 patients without at least one overnight ICU stay, the final cohort consisted of 2,742 unique patients (with 2,971 unique hospital admissions and 3,329 unique ICU stays) were included for analysis. These patients had a total of 15,645 bed days in the ICU (average ICU length of stay 4.7 days) and 32,008 bed days at the hospital (average hospital length of stay was 10.8 days). As detailed in Fig. 2, the study cohort consisted of patients aged between 18 and 91 years (mean 58.2 years, median 60 years, first and third quartiles 47–70 years). The cohort comprised 1,464 (53.4%) male patients and 1,278 (46.6%) female patients. A total of 324 (11.8%) of the patients were aged between 18 and 35 years, 459 (16.7%) were aged between 35 and 50 years, 883 (32.2%) were aged between 50 and 65 years, and 1,076 (39.3%) were aged above 65 years. No significant difference in ICU length of stay (LOS) was observed between the 18 to 65 age group (mean LOS 5.28 days) and the “65 + ” age group (mean LOS 5.24 days), with a *p* value of 0.5701. However, a significant difference was found between the sexes: female patients had a mean LOS of 4.53 days, whereas male patients had a mean LOS of 5.91 days (*p* < 0.001). From an initial set of clinical, demographic, and operational features, 162 clinically relevant features with sufficient data density (i.e., observed in at least 50 patients) were subsequently included in the ML models, as detailed in Tables 2 and 3.

As detailed in Table 6, among the 2,849 patients undergoing craniotomy, 2,742 (96.2%) were discharged after more than 24 h in the ICU, whereas only 107 (3.8%) were discharged within 24 h. The patients without at least one overnight ICU stay (*n* = 107) were excluded from the predictive models to ensure a focus on those with more extended postoperative monitoring (*n* = 2,742). However, a comparative analysis was conducted to highlight differences between the groups. While most procedural and diagnostic distributions did not reach statistical significance, one notable difference was observed in skull fractures. Among patients with an overnight ICU stay, the prevalence of skull fracture, head deformity, or traumatic brain injury was significantly lower (4.3%) compared with 11.1% in the short-stay group (*p* = 0.0027). This finding suggests that short-term ICU stays may be associated with observation for mild traumatic brain injury, particularly cases involving skull fractures, which may have been closely monitored before transitioning to standard inpatient care.

### ML model performances

**Fig. 3:**
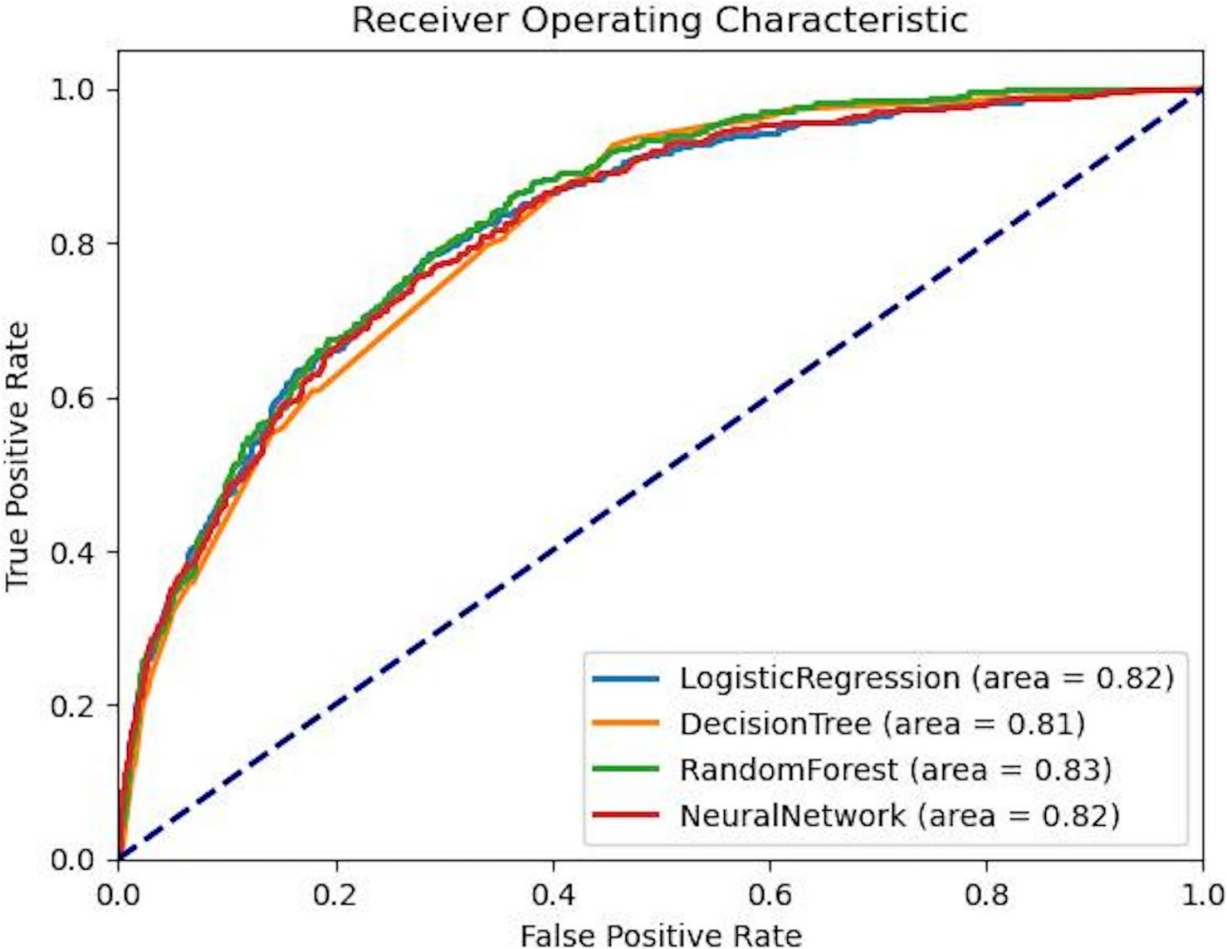
ROC Curve for Machine Learning Models Predicting ICU Discharge in Neurosurgery Patients

Figure 3 shows the receiver operating characteristic curve of all four models on the test sample, and Table 7 summarizes the performance of the four ML models evaluated across training, validation, and test samples. The RF model demonstrated the highest performance in terms of AUC on the training (0.861) and test samples (0.831) and was a close second on the validation sample (0.805). RF’s AP was 0.640, 0.535, and 0.561 on the training, validation, and test samples, respectively. Accuracy for the RF model was 0.831, 0.808, and 0.827 on the training, validation, and test samples, respectively. Finally, F1 scores were 0.383 (training), 0.288 (validation), and 0.339 (test). The NN model closely followed RF in predictive performance, with AUC values of 0.855 (training), 0.809 (validation), and 0.824 (test). The AP of the NN model reached 0.644 on the training sample and 0.558 on the test sample, with an accuracy of 0.830 on the test sample. The NN model’s F1 scores were 0.383 (training), 0.288 (validation), and 0.339 (test).

**Table 7:** Performances of machine learning models

Data Sets	Metrics	Machine Learning Methods			
		Logistic Regression	Decision Tree	Random Forest	Neural Network
Training	AUC	0.807	0.825	0.861	0.855
	Average Precision	0.563	0.544	0.640	0.644
	Accuracy	0.824	0.825	0.831	0.834
	F1 Score	0.424	0.451	0.383	0.433
Validation	AUC	0.799	0.793	0.805	0.809
	Average Precision	0.547	0.509	0.535	0.562
	Accuracy	0.818	0.812	0.808	0.823
	F1 Score	0.427	0.415	0.288	0.402
Test	AUC	0.821	0.813	0.831	0.824
	Average Precision	0.546	0.497	0.561	0.558
	Accuracy	0.829	0.822	0.827	0.830
	F1 Score	0.404	0.410	0.339	0.383

The LR outperformed DT among the interpretable models. LR achieved an AUC of 0.807 on the training sample and 0.821 on the test sample, an AP of 0.563 (training) and 0.546 (test), and an accuracy of 0.829 on the test sample. F1 scores for LR were 0.424 (training), 0.427 (validation), and 0.404 (test). In contrast, the DT model demonstrated slightly weaker performance, with an AUC of 0.825 (training) and 0.813 (test), an AP of 0.544 (training) and 0.497 (test), and an accuracy of 0.822 (test). The F1 scores for DT were 0.451 (training), 0.415 (validation), and 0.410 (test). These results highlight the superior predictive power of the RF and NN models, with interpretable models (LR and DT) providing stable, albeit slightly lower, performance across datasets.

### Feature importance (SHAP values)

**Fig. 4:**
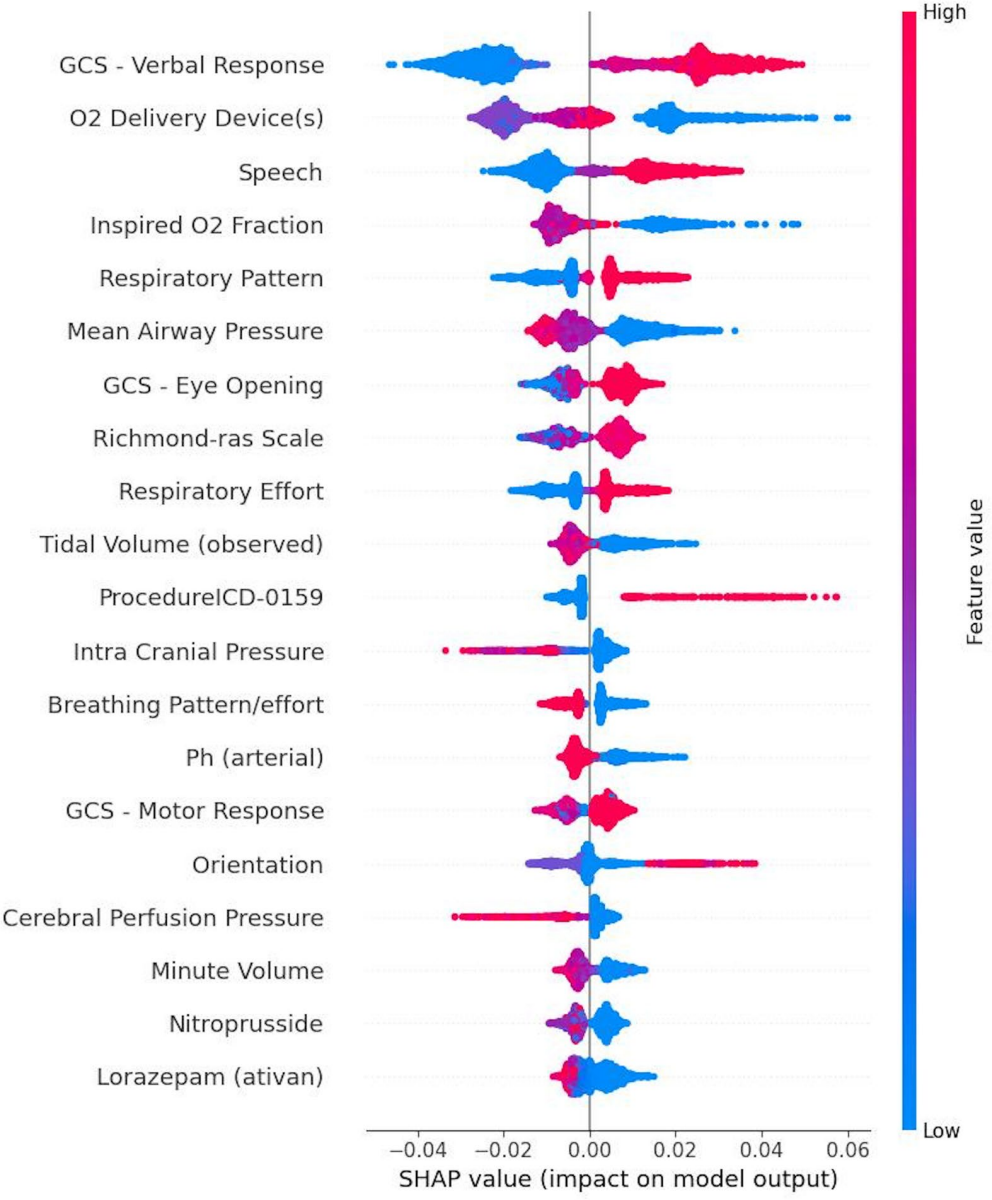
Feature Importance Based on SHAP Analysis for ICU Discharge Prediction

As summarized in Fig. 4, we conducted a standard SHAP explainability analysis on the RF model, which had the highest test sample AUC among the models tested in the previous section. Key predictors of SHAP analysis included GCS components (verbal response, eye opening, motor response), respiratory-related parameters (oxygen delivery devices, tidal volume, respiratory effort), intracranial pressure, arterial pH, and Richmond Agitation-Sedation Scale. These parameters were among the most essential predictors impacting ICU discharge. These features represent some of the key drivers identified by the SHAP analysis. As shown in Fig. 4, key predictors negatively impacting ICU discharge included respiratory-related parameters (e.g., low oxygen saturation, abnormal respiratory patterns) and high heart rate. Higher GCS scores, normal speech, and stable respiratory parameters were positive predictors.

## Discussion

Our study demonstrated that the RF and NN models outperformed the LR and DT models in predicting ICU discharge times for neurosurgery (craniotomy) patients within 24 h. The RF model achieved the highest AUC in the test sample (0.831), closely followed by the NN model. This indicates that both RF and NN models have solid predictive powers, with RF slightly leading in the overall performance. These models’ consistent and robust performance across all samples highlights their effectiveness in capturing the complex patterns necessary for accurate discharge predictions. The LR and DT models provided valuable insights into our analysis due to their interpretability and ease of use. They are beneficial in scenarios in which understanding the rationale behind predictions is essential. However, these models generally exhibited lower predictive success than the NN and RF models. The NN and RF models, often considered black-box due to their complex structures, demonstrated superior performance in terms of AUC across all samples.

Despite their complexity, the outcomes of these advanced ML models are not uninterpretable. By using SHAP analysis, as we did in this study, the predictions of these complex models can be explained, providing transparency in how the models make decisions. This approach allows clinicians to trust and understand the predictions these powerful yet complex models make, ensuring that they can be effectively integrated into clinical practice. In summary, although LR and DT models are more interpretable, the superior performance of NN and RF models can be harnessed effectively with the help of tools such as SHAP, combining the predictive power and interpretability.

Another critical aspect of our study was using a comprehensive set of clinical variables, many of which were initially recorded as verbal and subjective data. We converted these verbal examination datasets into numeric values, enabling the ML algorithms to process and analyze the data objectively (Table 2). This conversion process enhanced the accuracy and reliability of our predictive model and established a set of numeric metrics and scales that can be used in further studies. Standardized recording of verbal assessments using numeric scales can enhance the applicability of ML algorithms in predicting ICU discharge times and optimizing ICU resource management in neurosurgery patients.

Besides these approaches, we incorporated radiological report data extracted using a keyword and keyphrase-based approach to identify critical features such as midline shift, cerebral edema, and subdural hematoma. As detailed in Table 4, although these radiological findings provide valuable insights into patient conditions, their overall impact on the performance of ML models predicting ICU discharge was limited but led to a slight improvement. This suggests that although radiological indicators are clinically relevant, they may play a secondary role in influencing discharge outcomes compared with structured clinical and demographic features. The integration of radiological reports and ICD codes, as outlined in Tables 1 and 4, enriched the dataset and provided a more comprehensive assessment, contributing to a modest enhancement in model performance, even though the core predictive variables driving the results remained largely unchanged.

Research in this area underscores the promise of explainable ML techniques in enhancing discharge decision-making processes for various patient populations. Safavi et al. (2019) [11] developed and validated an ML algorithm to improve discharge processes for surgical inpatients. Their model decisively predicted 24-h discharge times with an AUC of 0.840, demonstrating the effectiveness of ML in estimating patient discharge times and its potential to enhance hospital organizations. The author’s ML algorithms results underscore the value of predictive analytics in resource planning and management. The authors also highlighted various barriers to discharge, including clinical complications, administrative delays, and patient-specific factors, which were systematically identified and incorporated into their model [11].

Khaniyev et al. (2023) [36] used a mixed-integer prescriptive optimization model alongside an ML algorithm to identify minimal barriers to patient discharge, effectively pinpointing high-yield barriers that are patient-specific and context-dependent. Similarly, Ward et al. (2021) developed a gradient-boosted model using hourly EMR data from multiple hospitals to predict patient discharge times, achieving an AUC of 0.729 and underscoring the importance of granular data in improving prediction accuracy [37]. Cuadrado et al. (2019) [14] developed an RF model to predict ICU discharge times, achieving a mean error of less than half a day with a coefficient of determination above 97%, highlighting the model’s precision in a high-stakes ICU environment. Zhang et al. (2021) [26] leveraged EMR access logs to enhance next-day discharge predictions, achieving an AUC of 0.921, illustrating the utility of detailed interaction data in discharge planning. McCoy et al. (2018) [38] assessed time-series ML methods for forecasting hospital discharge volumes, finding that ML methods performed well with high calibration (R^2^ values of 0.843 and 0.726 at two different sites), outperforming simpler carry-forward models in terms of accuracy and reliability. In comparison, our study focused on patients undergoing craniotomy in the ICU, a specific and complex patient cohort. We developed RF and NN models that achieved a higher AUC for all the datasets than the other two ML models. Our approach involved converting verbally recorded examination results into numeric values and scales, creating a standardized, objective metric system that enhanced the model’s accuracy and reliability.

In another notable study, the authors introduced a weak-supervision approach to predict prolonged hospital LOS, using a quantile regression model to define a flexible and principled LOS cutoff, allowing for effective classification despite unnecessary or unuseful labels [39]. Another study in this manner focused on predicting various aspects of patient discharges, including same-day and next-day discharges and hospital stay or mortality, with high accuracy (AUCs ranging from 0.819 to 0.964), demonstrating the applicability of interpretable ML models in a clinical setting to improve operational efficiency and patient care in major hospitals [17]. Levin et al. (2020) [8] implemented an ML-based discharge prediction model using real-time EMR data to support multidisciplinary discharge rounds, significantly reducing hospital LOS in certain units.

A recent study conducted a comprehensive review of 21 studies on the application of ML for predicting the LOS in medical inpatients, highlighting the variability in performance due to different input datasets, thresholds, and outcome metrics. The review identified common methodological shortcomings, such as insufficient reporting of patient demographics and clinical details, underscoring the need for standardized methodologies and prospective validation studies to enhance the utility of ML models in clinical settings [40]. This review is directly relevant to our research, highlighting the importance of comprehensive data collection and standardization to improve ML model accuracy and reliability, which we addressed by converting clinical observations into standardized numeric scales in our NN model.

Our study adds to this body of work by focusing specifically on patients undergoing craniotomy in the ICU, using an RF model that achieved superior AUC and accuracy values across all the datasets. Unlike previous studies, our approach involved converting verbally recorded clinical observations into standardized numeric scales, which enhanced the model’s accuracy and reliability. This method addressed the unique needs of a specialized patient cohort and provided a tailored solution that can be generalized across different hospital settings.

Future research should focus on prospective validation of the NN and RF models in multiple health care settings to ensure their generalizability and effectiveness across various patient populations. Integrating additional clinical variables and employing advanced modeling techniques could enhance predictive accuracy. Moreover, developing standardized protocols for converting verbal clinical observations into numeric scales across different institutions would mitigate bias and improve model reliability. Furthermore, incorporating multimodal data into these models presents a promising direction for future work. For instance, integrating radiology reports and other textual data with clinical variables could enrich the dataset, allowing the models to capture more complex and nuanced patient information. By leveraging the full spectrum of available data, including structured numerical data and unstructured text, future ML models could offer even more precise and actionable insights in the clinical setting. Collaborative efforts to create interoperable EMR systems can facilitate the seamless implementation of ML algorithms in clinical practice, ultimately improving ICU discharge processes and patient outcomes.

Our study has several limitations. Firstly, the dataset was derived from a single institution EMR, which may limit the generalizability of the findings to other healthcare settings. Additionally, although converting verbal clinical observations to numeric scales improved model accuracy, this process might introduce bias if not standardized across different clinical teams. Instead of keyword searches in radiological reports, more advanced foundational large language models could perform better in identifying desired features such as midline shift, hematoma, etc. The reliance on retrospective data also poses a limitation, as real-time application and prospective validation were not conducted within this study. Furthermore, the model’s performance might vary with different EMR systems and clinical workflows, which can be explored in a future study. Another limitation of our study is the potential influence of nonclinical factors, such as bed availability on the general ward, which may extend ICU stays despite patients being clinically ready for discharge. Although these factors were not directly included in our model due to data limitations, they represent an essential consideration for real-world applicability. Future studies incorporating administrative and logistical variables could further refine ICU discharge predictions.

## Conclusions

This study highlights the superior performance of RF and NN models in predicting ICU discharge times for neurosurgery patients, surpassing the more interpretable DT and LR models in predictive power. Although these advanced models typically face challenges in interpretability, tools such as SHAP allow us to maintain transparency, making these complex predictions actionable in clinical settings. A key aspect of our approach was the conversion of verbal clinical observations into standardized numeric values, which played a crucial role in enhancing the accuracy of the ML models. Moving forward, the validation of these methods across different clinical environments will be essential to confirm their utility and ensure their broad applicability.

## References

[1] Perez-Vega C, Sanghavi DK, Moreno Franco P, et al. Safety and feasibility of a fast-track pathway for neurosurgical craniotomy patients: bypassing the intensive care unit. Mayo Clin Proc Innov Qual Outcomes. 2023;7(6):534–43. 10.1016/j.mayocpiqo.2023.09.002. 38035051 10.1016/j.mayocpiqo.2023.09.002 PMC10685299

[2] Fugate JE. Complications of neurosurgery. CONTINUUM Lifelong Learn Neurol. 2015;21:1425–44. 10.1212/CON.0000000000000227 26426239

[3] Rao GSU, Muthuchellappan R. Neurologic emergencies after neurosurgery. In: *Essentials of Neurosurgical Anesthesia & Critical Care*. Springer International Publishing; 2020:501–506. 10.1007/978-3-030-17410-1_77

[4] Weng WH. Machine Learning for Clinical Predictive Analytics. Published online September 19, 2019.

[5] Sarwal A. Neurologic Complications in the Postoperative Neurosurgery Patient. CONTINUUM Lifelong Learn Neurol. 2021;27(5):1382–404. 10.1212/CON.0000000000001039 34618765

[6] De Grood A, Blades K, Pendharkar SR. A review of discharge-prediction processes in acute care hospitals. Healthc Policy. 2016;12(2):105–15. 28032828 PMC5221715

[7] Terwiesch C, Kc D, Kahn JM. Working with capacity limitations: operations management in critical care. Crit Care. 2011;15(4):308. 21892976 10.1186/cc10217 PMC3387581

[8] Levin S, Barnes S, Toerper M, et al. Machine-learning-based hospital discharge predictions can support multidisciplinary rounds and decrease hospital length-of-stay. BMJ Innov. 2021;7(2):414–21. 10.1136/bmjinnov-2020-000420.

[9] Yu R, Wang S, Xu J, et al. Machine learning approaches-driven for mortality prediction for patients undergoing craniotomy in ICU. Brain Inj. 2021;35(14):1658–64. 10.1080/02699052.2021.2008491. 35080996 10.1080/02699052.2021.2008491

[10] Aissaoui W, Khennou F, Abdellaoui A. Enhancing Intensive Care Patient Prognostics with Machine Learning. In: *Proceedings of the 12th International Symposium on Information and Communication Technology*. ACM; 2023:546–553. 10.1145/3628797.3628974

[11] Safavi KC, Khaniyev T, Copenhaver M, et al. Development and validation of a machine learning model to aid discharge processes for inpatient surgical care. JAMA Netw Open. 2019;2(12):e1917221. 10.1001/jamanetworkopen.2019.17221. 31825503 10.1001/jamanetworkopen.2019.17221 PMC6991195

[12] Mustafa A, Mahgoub S. Understanding and overcoming barriers to timely discharge from the pediatric units. BMJ Qual Improv Rep. 2016;5(1):3772. 10.1136/bmjquality.u209098.w3772. 10.1136/bmjquality.u209098.w3772 PMC5051419 27752313

[13] Ragavan MV, Svec D, Shieh L. Barriers to timely discharge from the general medicine service at an academic teaching hospital. Postgrad Med J. 2017;93(1103):528–33. 10.1136/postgradmedj-2016-134529. 28450581 10.1136/postgradmedj-2016-134529

[14] Cuadrado D, Riaño D, Gómez J, et al. Pursuing optimal prediction of discharge time in ıcus with machine learning methods. 2019:150–154. 10.1007/978-3-030-21642-9_20

[15] Cheng FY, Joshi H, Tandon P, et al. Using machine learning to predict ICU transfer in hospitalized COVID-19 patients. J Clin Med. 2020;9(6):1668. 10.3390/jcm9061668. 32492874 10.3390/jcm9061668 PMC7356638

[16] van Walraven C, Forster AJ. The TEND (Tomorrow’s expected number of discharges) model accurately predicted the number of patients who were discharged from the hospital the next day. J Hosp Med. 2018;13(3):158–63. 10.12788/jhm.2802. 29068440 10.12788/jhm.2802

[17] Bertsimas D, Pauphilet J, Stevens J, Tandon M. Predicting inpatient flow at a major hospital using interpretable analytics. Manuf Serv Oper Manag. 2022;24(6):2809–24. 10.1287/msom.2021.0971.

[18] Citerio G. Big data and artificial intelligence for precision medicine in the neuro-ICU: Bla, Bla, Bla. Neurocrit Care. 2022;37(S2):163–5. 10.1007/s12028-021-01427-6. 35023043 10.1007/s12028-021-01427-6 PMC9343268

[19] Megjhani M, Weiss M, Kwon SB, et al. Vector angle analysis of multimodal neuromonitoring data for continuous prediction of delayed cerebral ıschemia. Neurocrit Care. 2022;37(2):230–6. 10.1007/s12028-022-01481-8. 35352273 10.1007/s12028-022-01481-8 PMC11973884

[20] Hemphill JC. Pro: neurocritical care big data and Ai: it’s about expertise. Neurocrit Care. 2022;37(S2):160–2. 10.1007/s12028-021-01434-7. 35072924 10.1007/s12028-021-01434-7

[21] Dhar R, Meyfroidt G. Navigating the ocean of big data in neurocritical care. Neurocrit Care. 2022;37(S2):157–9. 10.1007/s12028-022-01558-4. 35799093 10.1007/s12028-022-01558-4

[22] Daghistani TA, Elshawi R, Sakr S, Ahmed AM, Al-Thwayee A, Al-Mallah MH. Predictors of in-hospital length of stay among cardiac patients: a machine learning approach. Int J Cardiol. 2019;288:140–7. 10.1016/j.ijcard.2019.01.046. 30685103 10.1016/j.ijcard.2019.01.046

[23] Thompson B, Elish KO, Steele R. Machine learning-based prediction of prolonged length of stay in newborns. In: *2018 17th IEEE International Conference on Machine Learning and Applications (ICMLA)*. IEEE; 2018:1454–1459. 10.1109/ICMLA.2018.00236

[24] Morton A, Marzban E, Giannoulis G, Patel A, Aparasu R, Kakadiaris IA. A comparison of supervised machine learning techniques for predicting short-term ın-hospital length of stay among diabetic patients. In: *2014 13th International Conference on Machine Learning and Applications*. IEEE; 2014:428–431. 10.1109/ICMLA.2014.76

[25] Naemi A, Schmidt T, Mansourvar M, Ebrahimi A, Wiil UK. Quantifying the impact of addressing data challenges in prediction of length of stay. BMC Med Inform Decis Mak. 2021;21(1):298. 10.1186/s12911-021-01660-1. 34749708 10.1186/s12911-021-01660-1 PMC8576901

[26] Zhang X, Yan C, Malin BA, Patel MB, Chen Y. Predicting next-day discharge via electronic health record access logs. J Am Med Inform Assoc. 2021;28(12):2670–80. 10.1093/jamia/ocab211. 34592753 10.1093/jamia/ocab211 PMC8633668

[27] Rajkomar A, Dean J, Kohane I. Machine learning in medicine. N Engl J Med. 2019;380(14):1347–58. 10.1056/NEJMra1814259. 30943338 10.1056/NEJMra1814259

[28] Muhlestein W, Akagi D, Chotai S, Chambless L. The impact of presurgical comorbidities on discharge disposition and length of hospitalization following craniotomy for brain tumor. Surg Neurol Int. 2017;8(1):220. 10.4103/sni.sni_54_17. 28966826 10.4103/sni.sni_54_17 PMC5609434

[29] Johnson ABLPTHSCLAMR. MIMIC-IV Clinical Database Demo. Physionet.org.

[30] Johnson AEW, Bulgarelli L, Shen L, et al. MIMIC-IV, a freely accessible electronic health record dataset. Sci Data. 2023;10(1):1. 10.1038/s41597-022-01899-x. 36596836 10.1038/s41597-022-01899-x PMC9810617

[31] Goldberger AL, Amaral LAN, Glass L, et al. PhysioBank, PhysioToolkit, and PhysioNet. Circulation. 2000;101(23):25. 10.1161/01.cir.101.23.e215 10851218

[32] Khaniyev T, Cekic E, Gecici NN, et al. Predicting mortality in subarachnoid hemorrhage patients using big data and machine learning: a nationwide study in Türkiye. J Clin Med. 2025;14(4):1144. 10.3390/jcm14041144. 40004675 10.3390/jcm14041144 PMC11856828

[33] Ribeiro MT, Singh S, Guestrin C. “Why Should I Trust You?” In: *Proceedings of the 22nd ACM SIGKDD International Conference on Knowledge Discovery and Data Mining*. ACM; 2016:1135–1144. 10.1145/2939672.2939778

[34] Lundberg S, Lee SI. A Unified Approach to Interpreting Model Predictions. In:; 2017.

[35] Lauritsen SM, Thiesson B, Jørgensen MJ, et al. The Framing of machine learning risk prediction models illustrated by evaluation of sepsis in general wards. NPJ Digit Med. 2021;4(1):158. 10.1038/s41746-021-00529-x. 34782696 10.1038/s41746-021-00529-x PMC8593052

[36] Khaniyev T, Copenhaver MS, Safavi KC, Levi R. A prescriptive optimization approach to identification of minimal barriers for surgical patients. Science. 2020;5:639. 10.1101/2023.03.24.23287694.

[37] Ward A, Mann A, Vallon J, Escobar G, Bambos N, Schuler A. Operationally-ınformed hospital-wide discharge prediction using machine learning. In: *2020 IEEE International Conference on E-Health Networking, Application & Services (HEALTHCOM)*. IEEE; 2021:1–6. 10.1109/HEALTHCOM49281.2021.9399025

[38] McCoy TH, Pellegrini AM, Perlis RH. Assessment of time-series machine learning methods for forecasting hospital discharge volume. JAMA Netw Open. 2018;1(7):e184087. 10.1001/jamanetworkopen.2018.4087. 30646340 10.1001/jamanetworkopen.2018.4087 PMC6324591

[39] Mann AJ, Bambos N. Weak-supervision for prolonged hospital length of stay prediction. In: *2022 IEEE International Conference on E-Health Networking, Application & Services (HealthCom)*. IEEE; 2022:199–204. 10.1109/HealthCom54947.2022.9982748

[40] Bacchi S, Tan Y, Oakden-Rayner L, Jannes J, Kleinig T, Koblar S. Machine learning in the prediction of medical inpatient length of stay. Intern Med J. 2022;52(2):176–85. 10.1111/imj.14962. 33094899 10.1111/imj.14962

